# Effects of the Human Gut Microbiota on Cognitive Performance, Brain Structure and Function: A Narrative Review

**DOI:** 10.3390/nu12103009

**Published:** 2020-09-30

**Authors:** Katie Louise Tooley

**Affiliations:** Cognition & Behaviour, Land Division (Edinburgh), Defence Science & Technology, Department of Defence, Edinburgh, SA 5111, Australia; Katie.Tooley@dst.defence.gov.au; Tel.: +61-8-7389-5373

**Keywords:** microbiota, gut-brain-microbiota axis, cognition, probiotic, synbiotic, prebiotic

## Abstract

Enhancing or preserving cognitive performance of personnel working in stressful, demanding and/or high tempo environments is vital for optimal performance. Emerging research suggests that the human gut microbiota may provide a potential avenue to enhance cognition. This review examines the relationship between the human gut microbiota, including modulators of the microbiota on cognition and/or brain function. For this narrative review, a total of *n* = 17 relevant human research items of a possible 1765 published between January 2010 and November 2018 were identified. Two overarching design methods for synthesis were observed: correlational or pre/post intervention. Limited correlational design studies linking microbiota to cognitive/brain structure endpoints existed (*n* = 5); however, correlations between microbiota diversity and enhanced cognitive flexibility and executive function were observed. Gut microbiota intervention studies to improve cognition or brain function (*n* = 12) generally resulted in improved cognition (11/12), in which improvements were observed in visuospatial memory, verbal learning and memory, and aspects of attentional vigilance. Limited studies were available to draw a detailed conclusion; however, available evidence suggests that gut microbiota is linked to cognitive performance and that manipulation of gut microbiota could be a promising avenue for enhancing cognition which warrants further research.

## 1. Introduction

Sustaining and enhancing cognitive performance and resilience for personnel (e.g., warfighters, first responders, emergency department staff) operating in challenging environments is vital for effective performance. It has been demonstrated that the microbiota confers many health benefits to the host which include protecting the gut, energy and nutrient absorption [1] and protection against viral diseases [2]. The gut microbiota is also known to be able to influence several factors including gut health, disease, inflammation and general health [3]. The previously understood bi-directional communication of the gut-brain axis consisted of communication via neural, endocrine and immune pathways but has now been extended to include the intrinsic link of the gut microbiota [4]. The microbiota releases molecules (metabolism bi-products or secretion of produced molecules) into the local environment which can mediate physiological changes within the brain via different modes of trafficking [5]. Further, it has been observed in humans (healthy and clinical populations) that differences in, or interventions of, the gut microbiota have translated into alterations in cognitive performance [6,7,8]. Thus, one promising pathway for improving cognitive processes/performance in people could be via the modulation of the human gut microbiota.

The bi-directional communication between the gut microbiota and the host can occur through a number of pathways. Specific bacteria have the capability of secreting molecules (bacteria-dependent) and stimulating epithelial and/or immune cells to release pro- or anti- inflammatory cytokines and thus alter immune function and reactivity [9]. Bi-directional communication with the central nervous system (CNS) is facilitated by the enteric (gut) nervous system via the Vagus nerve, where it has been demonstrated that vagal innervation, gut bacteria and brain behaviour are closely related [10]. Bacteria have the ability to communicate and cause change within the CNS through: (1) producing and secreting neurotransmitters in full or as metabolites/fractions, e.g., serotonin; and (2) secreting amino acids and other compounds including short-chain fatty acids and folate, or combinations thereof, which have the ability to communicate and cause change within the CNS [11,12]. However, the gut microbiota is sensitive to change and can therefore be influenced by many environmental factors including stress (sleep quality, chemicals, high-stress job), diet (healthy vs. unhealthy) and medication [13]. These external stressors can shift the microbiota profile in the direction of more non-preferential bacterial communities and/or alterations in bacterial diversity, known as ‘dysbiosis’, which has the potential to result in negative health effects [14,15]. Dysbiosis is known to cause increases in inflammatory markers, cytokines and metabolites, both locally and peripherally. Such changes have been linked to increased intestinal permeability and many diseases including inflammatory bowel disease, coeliac, allergy [16,17,18], and, more recently, psychological conditions such as anxiety [19] and depression [20].

The gut microbiota (healthy or dysbiotic) and its effect (causal relationship) on cognitive performance, or the linking of enhanced cognitive attribute(s) to a specific healthy microbiome (genomic and/or metabolomic) signature, has not been addressed in depth [21]. Most of this research to date has focused on mitigating stress, anxiety, mood and depression via manipulating or enhancing the gut microbiome [21]. The scope has more recently widened to include the influence on cognitive performance and/or brain function. Recent studies implementing gut microbiota interventions have demonstrated that specific products (single-species [22] or multi-species [6] probiotic, or prebiotic [23]) have the ability to interact with the brain and have elicited a positive bacteria-cognition relationship.

Organisations such as fire and ambulance services, police departments, hospitals and defence forces understand the importance of preparing and/or enhancing the cognitive performance of their personnel when required to operate in complex, taxing and stressful environments. Indeed, the increased stressors experienced worldwide in 2020 due to the COVID-19 pandemic could increase this scope to include the wider population in general. Specific to first responders, medical staff and warfighters, whether during training or high demand situations, exposure to stressors including sleep deprivation, suboptimal nutrition, physical or mental exhaustion/stress, or any combination thereof, are a common feature [15,24]. Indeed they have the potential to be costly or fatal to the individual or unit [25]. Decrements in cognitive performance are known to be the resultant effect of such stressors, but so is gut dysbiosis [24], thus the effects of demanding and stressful occupations may have a negative causal effect on the gut-microbiota-brain axis. Very little research has been conducted on personnel such as warfighters and first responders; however, cognitive measures/tasks relevant to such personnel have been examined. It is therefore possible that this knowledge could be applied in stressful and demanding contexts and that the gut microbiota of people such as warfighters and emergency services personnel could be manipulated such that it would translate to preserved/enhanced cognitive performance. We have recently reported on the usefulness of some dietary supplements [25] that may enhance cognitive performance. In an extension of this work to provide evidence-based information for potential interventions, it is important to understand if leveraging the human gut microbiota is another potential avenue. Thus, the aim of the current review is to better understand the interaction between the human gut microbiota and cognition, including the application of any nutritional intervention (probiotic, prebiotic, paraprobiotic or synbiotic) to determine what is currently known about enhancing cognition and/or brain functionality. Findings would inform both the wider community and those who operate in high stress environments, such as the modern warfighter. 

## 2. Materials and Methods

Preferred Reporting Items for Systematic Reviews and Meta-Analyses (PRISMA) methodology was generally adopted to aid with data synthesis; a scoping review focus was generally employed to aid in the ease of data synthesis. However, planning the design of this review commenced in August 2018, prior to the PRISMA extension for Scoping Reviews (PRISMA-ScR) as published in October 2018 [26]. As such, this review employed the five-step scoping review methodology previously proposed by Arskey and O’Malley [27] to assist in synthesis methodology and reporting of results. As this review is narrative in nature, it was not registered with the International Prospective Register of Systematic Reviews (PROSPERO). 

### 2.1. Identification and Screening of Relevant Studies

#### 2.1.1. Search Criteria

A wide electronic database search was conducted via SearchLight, a multidisciplinary search platform which accessed literature from Scopus, PubMed and PsycINFO for the purposes of this review. The following keywords were used for searching on 30 October 2018 for items listed from 2010 until the date of search. Search rules for a positive result included: required to have an item identified from “list 1” and also had to be paired with an item from “list 2” (See Table 1). Since cognition studies in this area can overlap with psychological conditions, additional terms were considered to ensure the widest capture of relevant data.

**Figure 1:**
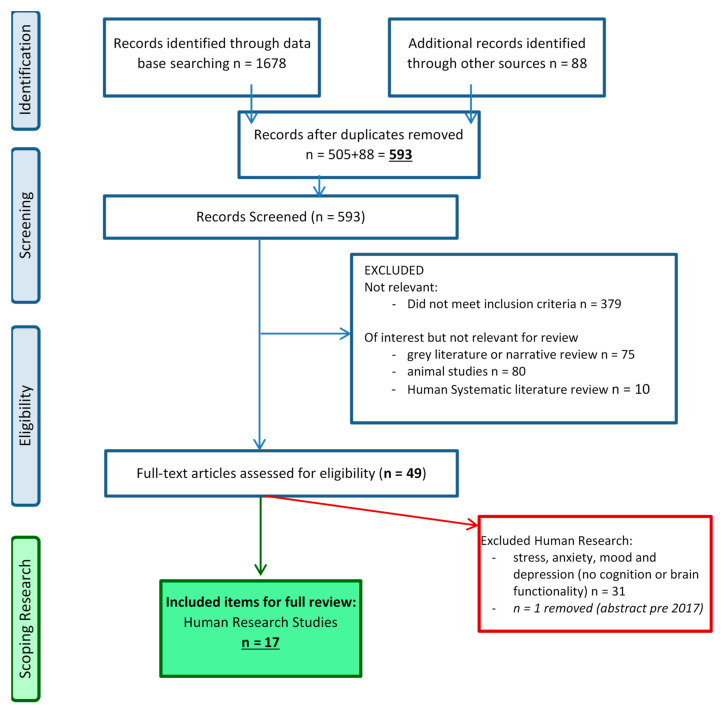
PRISMA 2009 search strategy Flow Diagram. Adapted from Moher et al.; The PRISMA Group (2009). Preferred Reporting Items for Systematic Reviews and Meta-Analyses: The PRISMA Statement. PLoS Med 6(7): e1000097. doi:10.1371/journal.pmed1000097. For more information, visit www.prisma-statement.org.

An initial pilot search query generated *n* = 80,000+ items, most of which contained largely unrelated data. Thus, the search criteria were redefined such that the “title” of the literature item had to meet the search criteria: a positive literature item had to have an item identified from “list 1”and from “list 2”. For further list refinement, a ‘does not include’ rule was incorporated which included the terms hepatic encephalopathy or Alzheimer. The requirement for search terms to be located in the article title meant that some known items were missed, as the paper keywords were not always in the title; therefore, as described by Moher et al. (2009), other known sources were included as represented in Figure 1 [28]. These included: reference lists from known published literature reviews, known existing networks, organisations, conferences and authors. These items were added manually for data synthesis. 

#### 2.1.2. Screening Process and Study Selection

The overall screening process can be viewed in Figure 1, adopting PRISMA inclusion/exclusion guidelines as previously described [28]. The refined search process, including manual and other searches, identified a total of *n* = 1766 items. Once duplicates had been removed, a total of *n* = 593 items remained for assessment. All items, irrespective of whether human or animal research were considered, as a list of animal research was to be kept for future reference. First pass screening assessed the “title only” (*n* = 225 excluded), followed by abstract scrutiny (second pass; *n* = 154 excluded) and full paper scrutiny. Reasons for exclusion included: not-relevant to inclusion criteria, non-English, unable to source. A more detailed explanation and full list of excluded items can be found in Supplementary Table S1.

The remaining items included for final pass screening (full manuscript; *n* = 214) were further collated into: human research; systematic literature review; animal research; and, finally, narrative review, blog, video or book (see Figure 1). Conference abstracts published during or after 2017 were included at the final review stage as it was deemed that an adequate amount of time may have passed to reach ‘full publication’ status. Conversely, abstracts published pre-2017 were excluded (inappropriate time-lapse to reach manuscript status). Focusing on human research only, an additional *n* = 165 items were collated and excluded separately as they were considered as items of interest for future reference. These items included: *n* = 80 animal research papers; *n* = 10 systematic literature reviews (with/without meta-analyses); and *n* = 75 narrative publications (reviews, books and grey literature). A total of *n* = 544 items were excluded throughout the process, leaving *n* = 49 items for full paper scrutiny.

#### 2.1.3. Data Extraction for Analysis

Endnote X8™ was used for synthesis and collation of data. During the ‘full paper scrutiny’ process, two obvious research design streams emerged: correlation (exploratory) and intervention(s). For research implementing an intervention, the type of intervention(s) was used as categories for discussion purposes and included: probiotic, prebiotic, synbiotic or paraprobiotic (no phytobiotic studies were found). These identified streams and sub-streams were used to compare data and report relevant findings. Additional information pertaining to type of trial, participant numbers, and the two overarching general findings were noted, and types of treatment were collected and charted for all items included for synthesis and comparison of findings. 

In the event that missing data or incorrect information was identified, the authors of the article were contacted to provide such information. In cases where contact was not successful, the description of the error has been recorded in the respective Table footer. Information pertaining to blinding, randomisation and appropriate control/placebo groups were captured in data synthesis tables. Omission of required detail was indicative of lower-quality papers or higher risk of bias.

## 3. Results

### 3.1. General Data Extraction and Matrices

Forty-nine research items were included for scrutiny (see Figure 1) and a further *n* = 31 were excluded due to their focus on stress, anxiety, mood and depression with no reference to cognitive or brain functionality measures; and finally a *n* = 1 conference abstract (pre 2017) was removed. This left *n* = 17 included papers for full review and synthesis. Specific to the warfighter, no military-population (or equivalent, e.g., emergency services) studies were identified, however, cognitive traits that are of interest were assessed.

### 3.2. Correlational Microbiota Design Studies

A total of *n* = 5/17 eligible studies were identified that focused on linking the microbiota and/or their metabolites to cognition or brain structure/function (as measured by functional Magnetic Resonance Imaging; fMRI) [12,29,30,31,32], see Table 2. The earliest published study (2015) explored the relationship between gut microbiota and brain structure and found distinct differences in brain microstructure parameters (i.e., hypothalamus, claudate nucleus and hippocampus) and that gut microbiota were able to differentiate between obese and non-obese people [29]. In addition, based on traill-making test scores, this study also found that an abundance of *Actinobacteria* was associated with better motor speed and attention, whereas increased numbers of *Prevotella* resulted in increased reaction time and poorer attention. Anderson et al. [30] performed an exploratory study and identified a positive association for gut microbiota with sleep quality and cognitive flexibility (Stroop Colour-Word test), where higher proportions of *Verrucomicrobia* and *Lentisphaerae* were linked to better cognitive flexibility in healthy older adults. A recent conference abstract (not yet published as a full manuscript) observed that a greater abundance of *Bacteriodetes* was linked to the ability of adult women (25–45 years) to maintain cognitive performance whilst experiencing increased task demand [31].

A recent study by Osadchiy et al. [12] in healthy males and females (18–60 years of age) demonstrated that faecal microbiota-derived (indole) metabolites were associated with functional and anatomical connectivity of the amygdala (a critical link to emotion) within the brain, which had a further association with obesity. Finally, it was observed that the abundance of the *Firmicutes* and *Bacteroidetes* families was correlated with structural brain alterations primarily found in the sensory integration areas and salience networks in patients with irritable bowel syndrome (IBS), with a distinct microbial profile compared to Healthy Controls (HC) [32].

### 3.3. Intervention Design Studies Linking Microbiota Profile(s)/Signatures to Cognition and Brain Structures/Function

Twelve original research articles were found where a gut microbiota intervention was used in an attempt to influence brain structures/function and/or cognition. Full details of these studies can be seen in Table 3. Interventions ranged from a single-species or multi-species probiotic, prebiotics or a paraprobiotic. Results have been addressed according to the types of intervention implemented.

#### 3.3.1. Probiotics

Seven studies used a probiotic intervention [6,7,8,22,33,34,35]. Positive effect(s) on cognition was observed in all studies apart from the study by Kelly et al. [33]. Three studies employed a single-species probiotic intervention [7,22,33], where three different single-species of bacteria (from either the *Bifidobacterium or Lactobacillus* genus) were assessed. Four studies implemented multi-species probiotic interventions and included bacteria from *Bifidobacteria*, *Lactobacilli*, *Lactococcus* or *Streptococcus* genera [6,8,34,35]. From a safety perspective, no significant medical side effects were observed. 

##### Single-Species Probiotic Intervention

Single species interventions ranged from 1 × 10^9^ to 2 × 10^10^ cfu/dose/day for a duration of 4 or 12 weeks. A small repeated-measures study (no cross-over; no randomisation) was carried out in healthy males (all consumed placebo (PLA) for four weeks then probiotic for four weeks) with daily ingestion at a relatively ‘low-dose’ (1 × 10^9^). Significant, but only considered as mild improvements, were observed in visuospatial memory, supported by enhanced frontal midline mobility (increased frequency) and a reduction in theta power, as measured by EEG [22]. Kelly et al. [33] implemented a probiotic (*Lactobacillus rhamnosus* (JB-1)) previously determined to have positive psycho-probiotic effects in animal models [40,41]. Employing a randomised, placebo-controlled, cross-over design (four weeks each) at a relatively ‘low-dose’ of 1 × 10^9^ cfu/dose/day, no positive effects on attention, executive function, memory, or emotional and social cognition were observed. Caution is required in interpretation of this study, as not only was a ‘low-dose’ administered but no “wash-out” period was employed, a known requirement for probiotic studies [42], increasing the likelihood of masking any acute effects that may have been evident. Interestingly, the multiple authors of this study were also co-authors of Allen et al. [22] where this ‘low-dose’ was successfully applied with a different probiotic using a repeated-measures design, different to that implemented in the previous study [33]. In a randomised, double-blind, placebo-controlled (RDBPC) study, 12 weeks of intervention (daily; 2 × 10^10^) with *Lactobacillus plantarum* improved verbal learning and memory as measured by the international shopping list memory test, in moderately stressed (as determined by Cohen’s Perceived Stress Scale) adults [7]. There were no further details pertaining to extra cognitive assessments.

##### Multi-Species Probiotic Intervention

Four studies utilised a multi-species probiotic intervention and employed a RDBPC study design [6,8,34,35]. Specific details pertaining to randomisation and/or blinding was not specified in two reports [6,34], which represented one large study split into two publications reporting on different aspects (see Table 3). Studies assessed the efficacy of a dose ranging from 6 billion to 12.5 billion revivable bacteria per day, consumed daily for a period of four–eight weeks. The first study published was by Tillisch et al., who investigated whether daily consumption of a five-species-containing probiotic for four weeks altered brain intrinsic connectivity or responses to emotional attention tasks [8]. It was observed that taking the probiotic daily for four weeks resulted in reduced activity at several brain regions, but most notably the sensory brain network area of the brain, controlling central processing for emotion and sensation, when compared to PLA or no-intervention. In addition, there were reductions in the intrinsic activity of the mid-brain resting-state, indicating changes in midbrain connectivity; these findings correlated to central processing and demonstrated for the first time that probiotics (this one specifically) affect gut-brain communication in humans. 

The two Bagga studies employed a probiotic stick containing nine different probiotic strains [6,34] at a dose of 7.5 × 10^9^ daily for four weeks. The 2018 Bagga et al. [6] study observed that the probiotic intervention improved cognitive reactivity and memory performance compared to PLA or nil intervention in healthy young adults. Further, it was found that these cognitive modalities were linked to brain signature differences and subtle differences in microbiome composition. In the 2019 Bagga et al. study [34] it was demonstrated that the probiotic intervention, when compared to PLA or nil intervention in healthy young adults, did not cause structural changes but resulted in significant changes to functional connectivity [34]. Several networks were impacted, but of most interest was the salience network, a large-scale brain network. The salience network, together with its interconnected networks, is important for modulating behaviour and improved attentional control associated with brain connectivity. Finally, a study performed in a clinical population with fibromyalgia by Roman et al. [35], demonstrated that daily ingestion of four-species probiotics for eight weeks improved the cognitive modalities of decision making and impulsivity compared to PLA control.

#### 3.3.2. Prebiotics

Two studies were identified using a prebiotic in an effort to modulate cognition [23,36]. Both studies were carried out in healthy adults. Neither study collected faecal samples to determine effects on the gut microbiota specifically; however, the prebiotics (fructo-oligosaccharide [FOS], Bimuno-galactooligosaccharide [B-GOS] or Oligofructose-Enriched-Inulin) employed for assessment represent those that are well-described within scientific literature and known to have an effect on gut microbiota, specifically *Lactobacilli* and *Bifidobacteria* [43,44,45,46]. Schmidt et al. [23] performed a RDBPC and assessed two different prebiotic fibres FOS or B-GOS, compared to PLA. FOS had no effect, but daily administration of B-GOS for three weeks improved attentional vigilance to positive and negative stimuli (as measured by the attentional dot-probe task) in the unmasked condition; i.e., a change in the emotional bias towards positive stimuli. Smith et al. [36] performed a cross-over study where acute effects of the prebiotic Oligofructose-Enriched-Inulin on multiple aspects of cognition were assessed four hours post ingestion. Whilst a prebiotic fibre was implemented in this study, due to the short time-frame in examining effect, this would be suggestive of a non-probiotic effect, as the intervention was ingested with food and gastrointestinal transit time to reach the large intestine would be greater than four hours. Interestingly, improvements in episodic memory (recall and recognition) were observed two h after ingestion, indicating that further assessment is required to determine the true causes for these changes.

#### 3.3.3. Paraprobiotics

Two studies implemented a paraprobiotic to determine the effect on cognition and brain behaviours [37,38]. As previously defined, paraprobiotics are a newer probiotic alternative as they are not ‘live’ bacteria and are therefore more stable from a consumer product point of view. They consist of non-viable microbial cells, cell fractions (wall, constituents), or crude cell extracts that have the ability to confer health benefits to the host [47]. A paraprobiotic treatment of *Lactobacillus helveticus* administered in tablet form at three different doses (500, 1000 or 2000 mg) compared to 0 mg-PLA for twelve weeks in healthy older adults (only *n* = 10/group) was performed by Chung et al. [37]. A variety of tests were used to assess cognition (memory, information processing, interference, vigilance); however, only minor improvements were observed for information processing accuracy in participants who received the low-dose (500 mg) intervention; all other assessments were not significant. A study by Ohsawa et al. [38] also employed *L. helveticus* as a paraprobiotic, but administered as a milk drink. This intervention also included a peptide fraction (lactononadecapeptide-19 amino acid peptide chain) derived from sour milk, which had been previously demonstrated to reduce memory deficits in mice [48,49]. In older adults with mild memory deficits, it was found that after eight weeks of intervention, improvements were seen in total neuropsychological status (as measured by the repeatable battery for assessment of neuropsychological status (RBANS) test score) and delayed memory pre- vs. post-intake within subjects; and attention and coding for pre- and post-intake within and between subjects.

#### 3.3.4. Synbiotic

No fully published research items were identified; however a recent study by Tooley et al. (conference abstract [39]; manuscript in preparation) assessed the effects of the synbiotic intervention ProGood™ on cognition in healthy young university students implementing a RDBPC designed study [39]. The synbiotic consists of two probiotic species at a total dose of 3 × 10^10^ cfu / 5 g and three types of prebiotic fibres (see Table 3 for full details). It was found that daily ingestion of 5 g dose of the synbiotic for four weeks resulted in improved immediate and delayed memory recall assessments compared to PLA-control. 

## 4. Discussion

### 4.1. Scope of Review

Enhancing or preserving cognitive performance of personnel operating in stressful and high-tempo environments is vital for optimal performance. The human gut microbiota is emerging as a potential biotechnological intervention to enhance cognitive performance. The overarching aim of this review was to better understand the interaction between the human gut microbiota and cognition, including the application of any intervention (probiotic, prebiotic, paraprobiotic or synbiotic) to determine what is currently known about enhancing cognition and/or brain functionality. Specifically, this was out-worked by considering two different approaches: (1) to determine if specific microbiota bacteria or microbiota ‘signatures’ (multiple bacteria genera) are linked to desirable brain functionality and cognition; and (2) to determine whether interventions of the gut microbiota translate to enhanced cognitive performance. No research specific to people working in demanding and stressful environments was identified for review. This review extends on previous published reports as it has collated and discussed relevant research performed in humans linking the gut to brain performance/function in populations commonly observed in healthy, stressed, anxious, moody or depressed (mood and mental well-being) individuals. Previous articles have specifically focused on one niche area and have included an array of other co-morbidities as part of their synthesis. This increases the complexity of results and reduces the applicability of the reported findings observed in relatively healthy people working in extreme environments, such as warfighters, police, emergency response personnel and/or relief aid workers. These findings could be further extended to include the general population, especially given the events of 2020 due to the COVID-19 pandemic and the subsequent stressors placed on individuals in everyday life. Interestingly, through the reviewing process it became apparent that no formal systematic or narrative literature review existed that specifically focused on and discussed the effects/links of the gut microbiota and cognition in humans. This narrative review has begun to address this critical knowledge gap.

### 4.2. Overall Synthesis

With respect to correlation (exploratory) studies, only five microbiota correlation studies that met the inclusion criteria were found to examine the link between gut microbiota and cognition/brain function. Significant relationships between microbiota diversity and enhanced cognitive flexibility and executive function were observed. Several bacterial phyla, including *Actinobacteria* and *Firmicutes*, which represent the genera *Bifidobacterium* and *Lactobacilli*, respectively, were linked; however, direct relationships at the level of specific family, genus or species (increased specificity) were not identified. Limited studies are available to draw definitive conclusions; however, these initial findings linking the gut microbiota to cognition are promising and warrant further research. Concerning the reviewed intervention studies, products shown to have a significant and meaningful effect have been identified for follow-up. These products include the prebiotic fibres B-GOS, trehalose, arabinogalactan and the probiotic bacteria *Lactobacillus acidophilus*, *Bifidobacterium lactis*, *Bifidobacterium longum* and *Bifidobacterium breve* (specific culture derivations described in Table 3). 

Generally, the studies were well designed; however, due to omission of critical design information, several studies would have been deemed as low-quality if GRADE and SIGN50 evaluations were to be performed. Most positive effects were linked to intervention containing a probiotic, a prebiotic, or a combination of the two (synbiotic). Most studies adopted a RDBPC design, and daily intervention ranged from a minimum four weeks (28 days) to twelve weeks, with the exception of the prebiotic studies (adopting the acute effect after four hours or a daily dose for three weeks). Dosing studies were not commonly performed. Given that formulation/type and derived-culture of the probiotic bacteria is critical information to determine the appropriate intervention dosage, it is difficult to specify a generic/average dose. Reviewed interventions to manipulate the microbiota generally led to positive effects in improving brain function and cognitive processes. Ten out of twelve studies observed marked improvements in at least one modality of cognition (one additional study with marginal improvement). Improvements were commonly seen in multiple aspects of memory (e.g., recall, recognition, visuospatial), verbal learning, emotional reactivity (fMRI), and attentional vigilance. Importantly, nil adverse effects of gut microbiota intervention were reported from all included studies. Most importantly, no negative effects on cognition and/or brain function were observed and interventions were generally well tolerated. Whilst this area of research is in its infancy and there is much heterogeneity between studies, the results are very promising and warrant further research.

### 4.3. Limitations

The studies included in the review are subject to various limitations. First, substantial variation was observed in sample size and composition. Sample size was variable from as low as *n* = 10 to 65/group; low sample sizes of *n* = 10 were identified in both correlational [20] and intervention [37] studies. Regarding composition, no studies (correlational or intervention design) were carried out in populations representative of personnel working in extreme environments, such as police, emergency services personnel and warfighters. 

Second, several issues were identified pertaining to research design. No longitudinal studies assessing the effect of job/life stress over time with/without an intervention were identified; in addition, the question of far-transfer duration has not been addressed. Dosing and duration of intervention studies were limited and derivation of proposed dosing schedule should be described. 

Third, intervention formulation was varied and studies were not repeated; in addition, complete studies comparing several treatment options did not exist and would be valuable for future research. This should be assessed within a longitudinal context. No studies were identified as determining a link between job/life stress (applied setting) on microbiota and job cognitive performance matrices and/or overall job performance.

Fourth addressing the link(s) to sleep hygiene (and circadian rhythms), nutritional intake, physical training and environmental stressors were commonly not accounted for. Knowing that these external stressors can alter the gut microbiota, these also need to be considered for interaction when analysing data. These metrics must be collected moving forward. 

Fifth, most intervention studies (11/12) did not examine the changes in the microbiota pre/post supplementation. Classification of the gut microbiota changes at the species level is critical when moving forward as such detail will inform appropriate formulation of gut-modulation treatments. 

Sixth, causal relationships of the gut microbiome on cognition in humans need to be better quantified via assays such as metabolomics and inflammatory matrices. Incorporation of such metrics will enable powerful retrospective and meta-analyses to be conducted in the future.

Finally, whilst no formal SIGN50 or GRADE evaluations were conducted for this narrative review, it was evident that several studies would be deemed as ‘low quality’ due to inadequate detail regarding randomisation, blinding techniques or inappropriate study design. 

### 4.4. Recommendations

Interestingly, whilst a number of limitations have been identified, either a positive relationship was identified or a positive cognitive effect was achieved via an intervention modulating the gut microbiota. Presently, there is a limited body of research in which varied gut microbiota interventions have been implemented to enhance cognition or brain function. As such, specific formal recommendations cannot be given at this stage. Also of interest, although not reported within this review, was the literature (see Figure 1 for reference and Supplementary Table S1 for list of all excluded items) that identified an association with the gut microbiota on stress, stress markers and anxiety. For this reason, a simple recommendation would be that more research needs to be performed in this area, specifically in healthy young adults exposed to stressful conditions.

### 4.5. Future Directions

Despite the limited number of studies, initial findings linking the gut microbiota to cognition are promising and warrant further research to address identified gaps and for application to the wider population. More specifically, pertaining to personnel operating in highly stressful environments, there is a need to: (1) examine the acute and chronic effects of military-relevant stressors on the gut microbiota of personnel such as warfighters and first responders; and (2) assess the efficacy of different types (novel and known) and/or combinations of gut microbiota interventions in relevant situations (for example, military and first responders). Further, additional scope for consideration should include wider assessment of the effects on mental health indices, physical performance and recovery, immune function/reactivity and inflammation, measurements of gut health and nutritional intake/processing. This review also identified research gaps that include: the lack of studies with military samples or on personnel working in extreme environments; the lack of longitudinal studies examining dosage effects and/or determining the duration of therapeutic effect; individual differences; and the efficacy of combined interventions. Indices that help inform how the gut microbiota influences the brain also need to be quantified in humans. These limitations should be addressed in future research. 

Provided that an adequate intervention dose is employed, daily intervention for a minimum of 28 days was demonstrated to be an effective duration for cognitive effects to be elicited and should be used as a minimum dosing duration moving forward. This proposed timeline represents potential improvements for cognitive effects only. For studies aiming to improve stress, anxiety and/or mood psychological metrics, consideration of an extended therapy/intervention duration or increased dosing may be required. Longitudinal studies need to be performed to determine far-reach efficacy, length of time to degradation, or if enhancements have a ‘solidified’ transfer. Probiotic cross-over studies are not recommended without consideration of two critical design criteria. These are: (1) the employment of an adequate wash-out period to ensure no residual effects of first treatment remain, which would be supported with evidence demonstrating ‘nil’ probiotic bacteria detected in faeces prior to cross-over and; (2) supporting evidence to demonstrate that cognitive ‘effect fade’ from the first treatment arm has indeed occurred. To circumnavigate the difficulty of these flaws, employing RDBPC designed studies would be more appropriate to reduce the risk of failure in cross-over studies due to inappropriate duration of ‘wash-out’ period [42]. 

## 5. Conclusions

It is important that personnel such as warfighters, first responders and hospital emergency personnel are able to display optimum performance in challenging and stressful environments. Despite no research specific to such personnel being identified, several studies reporting on relevant cognitive measures/tasks and their link to the gut microbiota within healthy/unhealthy populations were available for examination. A recently published narrative literature review authored by the US Department of Defense [50] included the discussion of evidence regarding the physical and/or cognitive benefits of probiotics in healthy adults. Their findings were broadly consistent with this completely independent narrative review. The authors concluded that despite the large number of ill-designed studies suggesting that there is no compelling evidence, there is promising evidence from the better designed studies that warrant further research. Further, it must be noted that overall findings would not be limited to warfighters or emergency services personnel and could be extended to inform the general population.

Future research efforts could benefit warfighters and emergency personnel, and the wider population at large, by (1) improving our understanding of microbiota signatures that are related to cognitive performance and job readiness, and (2) identifying appropriate gut microbiota interventions that support the optimal cognitive functioning and mental wellbeing of respective personnel. Overall, the available scientific evidence suggests that human gut-brain-microbiota axis is one avenue of research that could support enhancing or preserving warfighter cognitive performance. 

## Figures and Tables

**Table 1:** Search list items for literature review.

Search List 1		Search List 2
“Lactobacillus” or “Bifidobacterium” or “probiotic” or “prebiotic” or “psycho-biotic” or “microbiota” or “gut-brain-axis” or “gut microbiota” or “commensal bacteria” or “vaccae” or “lactobacilli” or “mycobacteria” or “immunomodulation” or “proinflammatory cytokine” or “gut permeability” or “microbial” or “microbiome” or “neurome”	**AND**	“cognition” or “cognitive” or “memory” or “vigilance” or “decision making” or “attention” or “visuo-spatial” or “executive function” or “task-switching” or “emotional” or “emotion” or “behaviour” or “behavior” or “recognition” or “resting-state” or “salience” or “anxiety” or “anxious” or “mood” or “moody” or “depression” or “depressive” or “PTSD” or “stroop” or “go-nogo” or “n-back” or “functional state” or “neuroscience” or “psychobiology”

**Table 2:** Exploratory human studies describing correlations/interactions between the gut microbiota and cognition, brain structure or function (no intervention).

Author/Year	Participants/Sample (± SD)	Sex (M/F)	Study Design	Assessment	Main Findings Microbiome Link
Fernandez-Real et al. (2015) [29]	*n* = 19 obese; *n* = 20 non-obese patients; total age range 30–65 years (mean ± SD not specified)	Not known	Correlational (partial blind); microbiome markers of obese vs. non-obese patients	fMRI; Trail making test; 16S rRNA gene sequencing	Specific phyla linked to obesity, brain structures and trail map making
Anderson et al. (2017) [30]	*n* = 37 healthy (50–85 years; 64.6 ± 7.5 years)	10/27	Correlational; microbiome, sleep quality and cognition	Stroop Colour-Word (cog flexibility); PSQI (sleep)	Association with sleep quality and cognitive flexibility
Taylor et al. (2017) [31]—conf. abstract	*n* = 34 (25–45 years old); no average +SD given in conference abstract	0/34	Correlational: microbiota and cognition	Modified flanker test	Greater numbers of *Bacteriodetes* = cog. performance maintained with increasing task demand
Osadchiy et al. (2018) [12]	*n* = 63 healthy adults (29.4 ± 10.8 years)	29/34	Correlational; microbiome metabolites and links to brain networks, obesity and anxiety	HAD; YFAS; MRI (structural, functional, diffusion); faecal metabolomics	Faecal metabolites linked to brain connectivity, reward networks, and anxiety symptoms
Labus et al. (2017) [32]	*n* = 29 IBS patients (26.1 ± 5.7 years); *n* = 23 HC (26.0 ± 6.5 years)	17/35	Correlational: microbiome markers in IBS and correlates of brain structure	HADs + PHQ-15; ETI-SR; PSS; compact MRI 16S rRNA gene sequencing	Behavioural link to microbiome in IBS: sensory and salience network regions, early-life trauma

Items listed in order of reference in text. Additional table data information: data were expressed as mean ± “value” but were not specified as standard deviation (SD) or standard error of the mean (SEM) in text. Healthy controls (HC); Pittsburgh Sleep Quality Inventory (PSQI); functional Magnetic Resonance Imaging (fMRI); Hospital Anxiety and Depression scale (HAD); Patient Health Questionnaire (PHQ-15); Early Trauma Inventory—Self Report (ETI-SR); perceived stress scale (PSS); magnetic resonance imaging (MRI); Yale Food Addiction Scale (YFAS); Irritable bowel syndrome (IBS).

**Table 3:** Human studies describing interventions on the gut microbiota and the effects on cognition, brain structures and function.

Author/Year	Participants/Sample (± SD)	Sex (M/F)	Study Design	Treatment	Dose/Frequency	Assessment	Main Findings—Microbiome Link
Probiotics							
Allen et al. (2016) [22]	*n* = 22 healthy males (22.5 ± 1.2 SEM y)	22/0	Repeated measures, placebo-controlled within-subject (blinding not stated)	*Bifidobacterium longum* 1714 strain	PRO = 1 × 10^9^ cfu/stick or PLA; 1 stick/day 4 weeks each. PLA→PRO	Cognitive tasks: CANTAB done with EEG	mild improvement vs. PLA in visuospatial memory; EEG profile consistent with improved memory
Kelly et al. (2017) [33]	Placebo-Probiotic group *n* = 15 (23.6 ± 1.0 year); Probiotic-Placebo group *n* = 14 (25.6 ± 1.1 year)	29/0	Randomised Placebo-controlled cross-over design (wash-out and randomisation not detailed)	*Lactobacillus rhamnosus* (JB-1)	Active treatment contained 1 × 10^9^ cfu/capsule; f = 1 daily 4 wk then cross-over	CANTAB	No improvement in cognitive parameters
Lew et al. (2018) [7]	Moderately stressed adults: *n* = 51/66 PLA (32.1 ± 11.4 year); *n* = 52/66 probiotic (31.3 ± 10.8 year)	12/39 12/40 (24/79)	RDBPC	*Lactobacillus plantarum* P8 (isolated from traditionally fermented sour milk—Mongolia)	2 g sachet of probiotic P8 or PLA P8 dose: 2 × 10^10^ cfu/day Daily 12 weeks	CogState Brief Battery	Social emotional speed response and verbal & memory learning improved; Cognitive and memory traits correlated with stress and anxiety. Sex different responses.
Tillisch et al. (2013) [8]	Females aged females (22.8 ± 2.7 year); *n* = 12 in fermented probiotic group, *n* = 11 in non-fermented control; *n* = 13 nil intervention	0/36	RDBPC (treatment, PLA and nil intervention)	Fermented milk containing *Bifidobacterium animalis* subsp *lactis* (strain number I-2494, *Streptococcus themophilus* and *Lactobacillus bulgaricus* (Danone Research Facilities)	*lactis* = 1.25 × 10^10^, *thermophilus* + *bulgaricus* = 1.2 × 10^9^; cfu/cup; f = daily 4 weeks	fMRI	affected activity of brain areas controlling central processing (emotion & sensation)
Bagga et al. (2018) [6]	Healthy volunteers: *n* = 15 no-intervention control (26.9 ± 5.0 year); *n* = 15 PLA (27.3 ± 5.8 year); *n* = 15 probiotic (28.3 ± 4.2 year)	7/8 9/6 7/8 (22/23)	RDBPC (randomisation and blinding not specified)	9 strains: *Lactobacillus casei* W56, *L. acidophilus* W22, *L. paracasei* W20, *Bifidobacterium lactis* W51, *L. salivarius* W24, *Lactococcus lactis* W19, *B. lactis* W52, *L. plantarum* W62 and *B. bifidum* W23	7.5 × 10^9^/3 g dose (*see extra table information*) vs. PLA or CON; f = daily 4 weeks	PANAS; SCL-90; ADS; LEIDS; fMRI with emotional decision making and recognition tasks	Microbiome composition mirrored self-reported behavioural measures and memory performance; potential link between specific *Bacteroides*, brain memory and recognition
Bagga et al. (2019) [34] - Epub May 2018	Healthy volunteers: *n* = 15 no-intervention control (26.9 ± 5.0 year); *n* = 15 PLA (27.3 ± 5.8 year); *n* = 15 probiotic (28.3 ± 4.2 year)	7/8 9/6 7/8 (22/23)	RDBPC (randomisation and blinding not specified)	See Bagga 2018 study	7.5 × 10^9^/3 g dose vs. PLA or CON; f = daily 4 weeks	fMRI	Changes in functional connectivity (link to depression and stress disorders) vs. PLA and CON
Roman et al. (2018) [35]	*n* = 40 fibromyalgia patients; complete study: probiotic *n* = 16/20 (55.0 ± 2.1 year); PLA *n* = 15/20 (50.3 ± 2.0 year)	1/15 2/13 (3/28)	Pilot RDBPC (blinding not specified)	ERGYPHILUS Plus (Laboratorios NUTERGIA, Spain): *Lactobacillus Rhamnosus* GG, *Lactobacillus Casei*, *Lactobacillus Acidophilus*, *Bifidobacterium Bifidus*.	6 × 10^9^/capsule (See Footnote) 2 capsules, twice daily; 8 weeks	Two-choice task and Iowa gambling task (impulsive choice and decision-making); mini mental state examination; urinary cortisol	probiotics improved impulsivity and decision-making in fibromyalgia patients
Prebiotics							
Schmidt et al. (2015) [23]	*n* = 15 PLA (23.3 ± 3.9 year); *n* = 15 FOS (24.5 ± 3.9 year); *n* = 15 B-GOS (23.3 ± 4.0 year)	7/8 8/7 7/8 (22/23)	RDBPC	Fructooligosaccharides (FOS) or Bimuno^®^-galacto-oligosaccharides (B-GOS)	5.5 g of FOS, B-GOS or PLA; Daily; 3 weeks	Attentional dot-probe task	B-GOS increased attentional vigilance to positive to negative stimuli
Smith et al. (2015) [36]	*n* = 47 (ave 23.0 years, range 19–30 years)	19/28	Cross-over (randomisation or blinding not detailed)	Oligofructose-Enriched Inulin or PLA added to de-caffeinated tea or de-caffeinated coffee	Pre-fasted 5 g prebiotic f = once0–4 h (acute effects)	Memory tasks; psychomotor tasks (simple reaction and selective attention tasks); sustained attention	Episodic memory tasks improved Psychomotor performance and selective attention unchanged.
Paraprobiotics							
Chung et al. (2014) [37]	Healthy adults *n* = 36/39: *n* = 10 PLA (64.5 ± 4.8 year); *n* = 10,500 mg (64.5 ± 2.2 year); *n* = 71,000 mg (64.43 ± 4.5 year); *n* = 92,000 mg (66.6 ± 5.0 year)	4/6 9/1 2/5 5/4 (20/16)	RDBPC (blinding not specified)	*Lactobacillus helveticus* (IDCC3801) Fermented (heat-treated) milk (LHFM); supernatant extracted and placed in tablet form.	took 4 tablets daily to reach a conc. of 500, 1000, 2000 or 0 mg (PLA) 12 weeks	Digit-span; Story recall; verbal learning; RVIP (cognitive fatigue measure); stroop; serial 3 s and 7 s	minor improvement in RVIP accuracy only for low dose of heat-treated fermented milk tablet
Ohsawa et al. (2018) [38]	All with mild memory deficits: *n* = 31/31 in fermented probiotic milk (58.5 ± 6.5 year); *n* = 29/30 PLA (57.8 ± 5.9 year)	13/18 13/16 (26/34)	RDBPC (blinding not specified)	Lactobacillus helveticus-fermented milk containing 2.4 mg lactononadeca-peptide (NIPPLTQTPV VVPPFLQPE). PLA contained no active ingredient	190 g drink with/without fermented peptide (2.4 mg) One daily 8 weeks	RBANS	Improvement in total RBANs and delayed memory score. Attention and coding score also improved. All other measures NS
Synbiotics							
Tooley et al. (2018) [39] - Conf abstract (manuscript in preparation)	Healthy young University Students: *n* = 34 Synbiotic; *n* = 33 PLA	16/51	RDBPC	*Lactobacillus acidophilus* L10 and *Bifidobacterium lactis* B94 plus arabinogalactan, inulin and trehalose	1.5 × 10^10^ of both bacteria strains cfu/5 g dose f = daily 4 weeks	Cognitive Battery	Synbiotic improved memory: immediate & delayed recall. Vigilance, attention, simple reaction time, executive control NS.

Items have been listed in order of discussion in main text. Placebo (PLA); Probiotic (PRO); electroencephalogram (EEG); Randomised, Double-blind, Placebo-controlled (RDBPC); colony forming units (cfu); Cambridge Neuropsychological Test Automated Battery (CANTAB; including motor screening, paired associates learning; attention switching, Rapid visual information processing (RVIP), emotion recognition and emotional Stroop); Positive and negative affect system (PANAS); Symptoms checklist-90 (SCL-90); Allgemeine Depressionskala (German; ADS); Leiden index of depression severity (LEIDS); Repeatable Battery for the Assessment of Neuropsychological Status (RBANS; comprises 12 subtests that contribute to one of five indexes: immediate memory, visuospatial/constructional, language, attention and delayed memory). not significant (NS). Note: A suspected error of dosage (stated in-text as × 10^6^) was identified in three studies [6,34,35]. Comparison of product online indicated error, author contact was pursued. Successful author contact was achieved for only one item [35], and the error was confirmed; correct dose was 6 × 10^9^. No response from [6,34], thus dose listed online was deemed correct and the defined dose stated within the manuscripts were deemed as typographical errors.

## Supplementary Materials

The following are available online at https://www.mdpi.com/2072-6643/12/10/3009/s1, Table S1: Excluded papers and reasons for exclusion.
