# Supplementary material for: Effects of the Human Gut Microbiota on Cognitive Performance, Brain Structure and Function: A Narrative Review
Source: Nutrients. 2020 Sep 30;12(10):3009. doi: 10.3390/nu12103009 (PMC7601389; doi:10.3390/nu12103009)
Supplement: Supplementary file 1 [file nutrients-12-03009-s001.pdf]

nutrients MDPI

Supplementary Table S1. List of all excluded items describing point of exclusion and reason

Author	Year	Experiment / Review	Type<br>Human (H), animal. (A), n/a, or unknown (?)	Excluded Y/N<br>(Level of exclusion and reason)
- Chronic helminth infection	2018	R	?	Y – Title – Not relevant
Ye, L. et al. (erratum)	2014	E	A	Y – Title – Not relevant
- probiotic treatment (Engineering news)	2013	?	?	Y – Title – Not relevant
Aagaard et al.	2016a	E	A	Y – Title – Not relevant
Aagaard et al.	2016b	E	A	Y – Title – Not relevant
Abdal.lah et al.	2018	E	n/a	Y – Title – Not appropriate
Aitbal.i et al.	2018	E	A	Y – Title – Not relevant
Akouche et al.	2016	E	n/a	Y – Title – Not appropriate
Al.cock et al.	2014	R	?	Y – Title – Not appropriate
Al.exeev et al.	2017	E	A	Y – Title – Not relevant
Al.ifirova et al.	2016	E	H	Y – Title – Not relevant
Anderson et al.	2016	R	?	Y – Title – Not appropriate
Armstrong et al.	2018	E	n/a	Y – Title – Not appropriate
Arseneault-Bréard et al.	2012	E	A	Y – Title – Not appropriate
Atia et al.	2018	E	n/a	Y – Title – Not appropriate
Ayres	2016	R	?	Y – Title – Not appropriate
Azpiroz et al.	2017	E	H	Y – Title – Not relevant
Bachem et al.	2017	E	n/a	Y – Title – Not appropriate
Badri et al.	2013	E	A	Y – Title – Not relevant
Bal.l et al.	2018	E	A	Y – Title – Not relevant
Bambling et al.	2017	E	H	Y – Title – Not relevant
Beilharz et al.	2016	E	A	Y – Title – Not relevant
Belkaid et al.	2016	E	n/a	Y – Title – Not appropriate
Blunt et al.	2015	E	n/a	Y – Title – Not appropriate
Bokulich et al.	2016	E	n/a	Y – Title – Not appropriate
Borrelli et al.	2016	E	A	Y – Title – Not relevant
Bradshaw et al.	2014	E	H	Y – Title – Not relevant
Bruce-Keller et al.	2017	E	A	Y – Title – Not relevant
Caroll et al.	2018	E	H	Y – Title – Not relevant
Casotti et al.	2017	E	H	Y – Title – Not relevant
Chen, K et al.	2017	E	?	Y – Title – Not appropriate
Cho, N. et al.	2017	E	A	Y – Title – Not relevant
Chrobak et al.	2016	R	H	Y – Title – Not relevant
Chunchai et al.	2018	E	A	Y – Title – Not relevant
Coleman et al.	2014	R	n/a	Y – Title – Not appropriate
Comstock et al.	2017	E	A	Y – Title – Not relevant
Corpuz et al.	2018	E	A	Y – Title – Not relevant
D'Mello et al.	2014	E	n/a	Y – Title – Not appropriate
Da Sliva et al.	2012	E	n/a	Y – Title – Not appropriate
Dal.lal. et al.	2012	E	n/a	Y – Title – Not appropriate
Daulatzai	2014	R	?	Y – Title – Not appropriate
Davis et al.	2016	E	A	Y – Title – Not relevant
Desbonnet et al.	2010	E	A	Y – Title – Not relevant

**Author**	**Year**	**Experiment / Review**	**Type** Human (H), animal. (A), n/a, or unknown (?)	**Excluded Y/N** (Level of exclusion and reason)
Diviccaro et al.	2019	E	A	Y – Title – Not relevant
Echeverry-Al.zate et al.	2017	E	?	Y – Title – Not appropriate
Eisele et al.	2018	E	H	Y – Title – Not relevant
El-Dieb et al.	2012	E	n/a	Y – Title – Not appropriate
El-Kholy et al.	2014	E	n/a	Y – Title – Not appropriate
Elder & Glickman-Simon	2013	R	?	Y – Title – Not appropriate
Engel et al.	2015	E	?	Y – Title – Not appropriate
Fakri et al.	2016	E	n/a	Y – Title – Not appropriate
Famodu	2016	E	H	Y – Title – Not relevant
Fasano	2017	R	?	Y – Title – Not appropriate
Feng, X. et al.	2017	E	A	Y – Title – Not relevant
Fernandes et al.	2013	E	n/a	Y – Title – Not appropriate
Fernando et al.	2018	E	n/a	Y – Title – Not appropriate
Fetissov	2017	R	A	Y – Title – Not relevant
Fleming et al.	2017	E	A	Y – Title – Not relevant
Fonken et al.	2018	E	A	Y – Title – Not relevant
Forsatkar et al.	2017	E	A	Y – Title – Not relevant
Forsythe & Bienenstock	2016a	R	?	Y – Title – Not appropriate
Forsythe & Bienenstock	2016b	R	?	Y – Title – Not appropriate
França & Lotti	2017	R	H	Y – Title – Not relevant
Fukudo	2016	?	?	Y – Title – Not appropriate
Gaetani	2011	?	?	Y – Title – Not appropriate
Gato et al.	2018	R	H	Y – Title – Not relevant
Geirnaert et al.	2013	E	n/a	Y – Title – Not appropriate
Ghita et al.	2015	E	n/a	Y – Title – Not appropriate
Gilbert et al.	2013	E	A	Y – Title – Not relevant
Go, J. et al.	2016	E	A	Y – Title – Not relevant
Grant & Baker	2017	R	A+H	Y – Title – Not relevant
Groff et al.	2018	E	A	Y – Title – Not relevant
Griffiths & Kikafunda	2015	?	H	Y – Title – Not relevant
Gu, C. et al.	2017	E	A	Y – Title – Not relevant
Gu, F. et al.	2018	E	n/a	Y – Title – Not appropriate
Guernier et al.	2017	?	H	Y – Title – Not relevant
Hassan. A.M. et al.	2017	E	A	Y – Title – Not relevant
Hassan. A.M. et al.	2018	E	A	Y – Title – Not relevant
Heijtz	2016	R	?	Y – Title – Not appropriate
Heintz-Buschart et al.	2018	E	H	Y – Title – Not relevant
Hernandez et al.	2018	E	A	Y – Title – Not relevant
Heydari et al.	2018	E	n/a	Y – Title – Not appropriate
Heyde & Ruder	2015	E	n/a	Y – Title – Not appropriate
Hoffman et al.	2017	E	?	Y – Title – Not appropriate
Hoggard et al.	2018	R	?	Y – Title – Not appropriate
Hoisington et al.	2018	R	?	Y – Title – Not appropriate
Houdek	2018	R	?	Y – Title – Not appropriate
Hu, S. et al.	2018a	E	H	Y – Title – Not relevant
Hu, S. et al.	2018b	E	H	Y – Title – Not relevant
Huang, S.-Y. et al.	2018	E		Y – Title – Not relevant
Ibrahim et al.	2018	E	A	Y – Title – Not relevant

**Author**	**Year**	**Experiment / Review**	**Type** Human (H), animal. (A), n/a, or unknown (?)	**Excluded Y/N** (Level of exclusion and reason)
Jang, S.-E. et al.	2011	E	A	Y – Title – Not relevant
Jenkins et al.	2016	R	?	Y – Title – Not appropriate
Jeong, J.-J. et al.	2015a	E	A	Y – Title – Not relevant
Jeong, J.-J. et al.	2015b	E	A	Y – Title – Not relevant
Jones, M.B. et al.	2017	E	n/a	Y – Title – Not appropriate
Kaban et al.	2010	E	n/a	Y – Title – Not appropriate
Kaczmarek et al.	2017	E	?	Y – Title – Not appropriate
Kadowaki et al.	2047	?	?	Y – Title – Not appropriate
Kahl et al.	2018	R	?	Y – Title – Not appropriate
Kang, S.S. et al.	2014	?	?	Y – Title – Not appropriate
Kao, A. et al.	2018	?	?	Y – Title – Not appropriate
Kargozari et al.	2015	E	n/a	Y – Title – Not appropriate
Kelly, J.R. et al.	2016	E	A	Y – Title – Not relevant
Kelsey et al.	2017	E	A	Y – Title – Not relevant
Kennedy et al.	2016	E	H	Y – Title – Not relevant
Khem et al.	2016	E	n/a	Y – Title – Not appropriate
Kim, J.S. & La Serre	2018	R	?	Y – Title – Not appropriate
Kleiman et al.	2015	E	H	Y – Title – Not relevant
Knudsen et al.	2017	?	?	Y – Title – Not appropriate
Kraimi et al.	2018	E	A	Y – Title – Not relevant
Lach et al.	2018	R	?	Y – Title – Not appropriate
Lakhtin et al.	2017	E	H	Y – Title – Not relevant
Lawrence & Hyde	2017a	E	H	Y – Title – Not relevant
Lawrence & Hyde (retracted)	2017b	E	H	Y – Title – Not relevant
Leclercq et al.	2017	E	A	Y – Title – Not relevant
Leitão-Gonçal.ves et al.	2017	E	A	Y – Title – Not relevant
Leung & Thuret	2015	R	?	Y – Title – Not appropriate
Lewis et al.	2017	E	H	Y – Title – Not relevant
Lim et al.	2017	E	A	Y – Title – Not relevant
Liu, K. & He, R.	2017	R	?	Y – Title – Not appropriate
Liu, Y. et al.	2016	E	H	Y – Title – Not relevant
López, P. et al.	2018	E	A	Y – Title – Not relevant
Lu, J. et al.	2018	E	A	Y – Title – Not relevant
Luna, R.A.	2018	E	H	Y – Title – Not relevant
Lv, F. et al.	2017	E	H	Y – Title – Not relevant
Magal.haes et al.	2017	E	A	Y – Title – Not relevant
Mahmoud et al.	2018	E	A	Y – Title – Not relevant
Manderino et al.	2017	E	H	Y – Title – Not relevant
Mangiola et al.	2016	R	?	Y – Title – Not appropriate
Manook et al.	2016	?	?	Y – Title – Not appropriate
Marques Ribeiro et al.	2012a	?	?	Y – Title – Not appropriate
Marques Ribeiro et al.	2012b	?	?	Y – Title – Duplicate
Massaro Jr et al.	2013	E	A	Y – Title – Not relevant
Meyrel et al.	2018	R	?	Y – Title – Not appropriate
Mi, G.L. et al.	2015	E	H	Y – Title – Not relevant
Mika et al.	2017	E	A	Y – Title – Not relevant
Moeller et al.	2016	E	A	Y – Title – Not relevant
Moerkl et al.	2017	E	H	Y – Title – Not relevant

**Author**	**Year**	**Experiment / Review**	**Type** Human (H), animal. (A), n/a, or unknown (?)	**Excluded Y/N** (Level of exclusion and reason)
Moloney et al.	2014	R	?	Y – Title – Not appropriate
Moraes Correia et al.	2014	E	n/a	Y – Title – Not appropriate
Muzny et al.	2013	E	H	Y – Title – Not relevant
Nicola et al.	2016	?	?	Y – Title – Not appropriate
Nunez et al.	2018	E	A	Y – Title – Not relevant
O'Hagan	2017	E	A	Y – Title – Not relevant
Orikasa et al.	2016	E	A	Y – Title – Not relevant
Pace et al.	2018	E	A	Y – Title – Not relevant
Painold et al.	2018	E	H	Y – Title – Not relevant
Pal.omba et al.	2012	E	n/a	Y – Title – Not appropriate
Panee et al.	2018	E	H	Y – Title – Not relevant
Park, S. et al.	2016	E	A	Y – Title – Not relevant
Parois et al.	2017	E	A	Y – Title – Not relevant
Patra et al.	2018`	E	n/a	Y – Title – Not appropriate
Pyndt Jørgensen	2015a	E	A	Y – Title – Not relevant
Pyndt Jørgensen	2015b	E	A	Y – Title – Not relevant
Qu, Y. et al.	2017	E	A	Y – Title – Not relevant
Raulo et al.	2018	E	A	Y – Title – Not relevant
Reininghaus et al.	2018	E	H	Y – Title – Not relevant
Reis Scheider et al.	2018	E	A	Y – Title – Not relevant
Rezaei et al.	2014	E	n/a	Y – Title – Not appropriate
Rodriguez, M. et al.	2016	R	?	Y – Title – Not appropriate
Rodriguez, O. et al.	2011	E	n/a	Y – Title – Not appropriate
Roman, P. et al.	2018a	R	?	Y – Title – Not appropriate
Roman, P. et al.	2018b	E	H	Y – Title – Not relevant
Ross, S.M	2017	R	?	Y – Title – Not appropriate
Ruchal.la, E.	2017	?	?	Y – Title – Not appropriate
Saghafian-Hedengren et al.	2018	R	H	Y – Title – Not relevant
Sal.mazadeh et al.	2018	E	?	Y – Title – Not appropriate
Santos, W.P. et al.	2017	E	A	Y – Title – Not relevant
Schembre et al.	2016	E	H	Y – Title – Not relevant
Schnorr & Bachner	2016	R	?	Y – Title – Not appropriate
Schoemaker et al.	2018	E	A	Y – Title – Not relevant
Selkrig et al.	2018	E	n/a	Y – Title – Not appropriate
Seratlić et al.	2013	E	n/a	Y – Title – Not appropriate
Sharma & Kanwar	2018	E	n/a	Y – Title – Not appropriate
Shen, H	2015	R	?	Y – Title – Not appropriate
Siddiqui & Harvey	2013	R	?	Y – Title – Not appropriate
Silva et al.	2011	E	n/a	Y – Title – Not appropriate
Sim, H.L et al.	2013	E	n/a	Y – Title – Not appropriate
Simko	2017	R	?	Y – Title – Not appropriate
Siwek et al.	2017	E	H	Y – Title – Not relevant
Soltani et al.	2018	E	n/a	Y – Title – Not appropriate
Stevens, B.R. et al.	2018	E	H	Y – Title – Not relevant
Stokes, C.S. et al.	2015	E	H	Y – Title – Not relevant
Su, A. et al.	2018	E	A	Y – Title – Not relevant
Sun, J et al.	2018	E	A	Y – Title – Not relevant
Sylvia et al.	2017	E	A	Y – Title – Not relevant

**Author**	**Year**	**Experiment / Review**	**Type** Human (H), animal. (A), n/a, or unknown (?)	**Excluded Y/N** (Level of exclusion and reason)
Szymanska-Czerwinska & Bednarek	2011	E	A	Y – Title – Not relevant
Tanriover et al.	2013	R	?	Y – Title – Not appropriate
Tarr, A. et al.	2015	E	A	Y – Title – Not relevant
Togher et al.	2017	E	H	Y – Title – Not relevant
Togher et al.	2017	E (abstract)	H	Y – Title – Not relevant
Toyoda et al.	2018	E	A	Y – Title – Not relevant
Trivedi, M et al.	2017	R	?	Y – Title – Not appropriate
Turpin et al.	2013	E	A	Y – Title – Not relevant
Val.-Laillet	2017	E	A	Y – Title – Not relevant
Vanhoecke et al.	2016	E	n/a	Y – Title – Not appropriate
Vázquez, G. H. et al.	2017	R	H&A	Y – Title – Not relevant
Vijayendra & Gupta	2013	E	n/a	Y – Title – Not appropriate
Wang, G et al. 2018	2018	E	A	Y – Title – Not relevant
Wang, J et al.	2016	? (abstract)	?	Y – Title – Not appropriate
Wang, W.C. et al.	2018	E	A	Y – Title – Not relevant
Watson Ronal.d	2015	?	?	Y – Title – Not appropriate
Waworuntu et al.	2017	E	?	Y – Title – Not appropriate
Webb, B et al.	2017	E (abstract)	?	Y – Title – Not appropriate
Wessells et al.	2017	E	H	Y – Title – Not relevant
Woo, J.Y. et al.	2014	E	A	Y – Title – Not relevant
Woods, J.A. et al.	2016	E	A	Y – Title – Not relevant
Xiao, H.-w. et al.	2018	E	A	Y – Title – Not relevant
Xiao, J. et al.	2014	E	A	Y – Title – Not relevant
Ye, L. et al.	2014	E	A	Y – Title – Not relevant
Yu, M. et al.	2017	E	H	Y – Title – Not relevant
Zal.ar, B et al.	2018	R	H	Y – Title – Not relevant
Zanette et al.	2015	E	n/a	Y – Title – Not appropriate
Zhao, X.H. et al.	2012	E	n/a	Y – Title – Not appropriate
Zhuikova et al.	2017	E	n/a	Y – Title – Not appropriate
Аблеев, Д. Р	2018	?	?	Y – Title – Not English
Алифирова, В. М.	2016	?	?	Y – Title – Not English
سحرفرزانه,	2018	?	?	Y – Title – Not English
刘国荣, 任桂美, 李, 雪.,	2018	?	?	Y – Title – Not English
徐, 娜., 李聪, 潘智华,	2018	?	?	Y – Title – Not English
psychobiotic-revolution.com		?	grey	Y – Abstract – Not appropriate
Webpage: neutraceutical.s World	2018	?	?	Y – Abstract – Not appropriate
Abildgaard, A. et al.	2014	E (abstract)	A	Y – Abstract – animal. <2017
Agusti et al.	2018	?	?	Y – Abstract – obesity
Al.len, A.P. et al.	2016a	E (abstract)	H	Y – Abstract – no abstract
Al.len, A.P. et al.	2016b	E (abstract)	H	Y – Abstract – no abstract
Anglin et al.	2014	E (abstract)	H	Y – Abstract – no abstract
Anglin et al.	2016	E (abstract)	H	Y – Abstract – no abstract
Arslanova et al.	2018	E (abstract)	A	Y – Abstract – antibiotic int.
Avila-Nava et al.	2017	E (abstract)	A	Y – Abstract – no abstract
Berg et al.	2015	E	A	Y – Abstract – Not relevant

**Author**	**Year**	**Experiment / Review**	**Type** Human (H), animal. (A), n/a, or unknown (?)	**Excluded Y/N** (Level of exclusion and reason)
Biron et al.	2014	R	?	Y – Abstract – Not relevant
Braniste et al.	2014	E	A	Y – Abstract – Not relevant
Buffington et al.	2016	E	A	Y – Abstract – Not relevant
Burnet P	2015	E (abstract)	?	Y – Abstract – Not appropriate
Cal.arge, C	2016	E (abstract)	?	Y – Abstract – Not appropriate
Cal.laghan, B et al.	2016	E	A	Y – Abstract – Not relevant
Carlson, A et al.	2016	E	?	Y – Abstract – Not relevant
Carlson, A et al.	2018	E	H	Y – Abstract – Not relevant
Clarke & Cyran	2016	R	?	Y – Abstract – Not appropriate
Clarke, G. et al.	2012a	?	?	Y – Abstract – Not appropriate
Clarke, G. et al.	2012b	?	?	Y – Abstract – Not appropriate
Clarke, G. et al.	2014	R	Al.l?	Y – Abstract – post natal.
Cowan, C et al.	2016	E	A	Y – Abstract – Not relevant
Craven, L et al.	2017	E	A	Y – Abstract – Not relevant
Cryan, J.	2014	R	?	Y – Abstract – Not appropriate
Cryan, J.F.	2016	R (abstract)	A&H	Y – Abstract – Not appropriate
Cryan, J	2018	R (abstract)	A&H	Y – Abstract – early develmt
Cryan & Dinan	2014	?	?	Y – Abstract – no abstract
Cryan & Dinan	2015	?	?	Y – Abstract – no abstract
Cryan & Dinan	2015	R (abstract)	A&H	Y – Abstract – early develmt
Davari, S et al.	2012	E	A	Y – Abstract – Not relevant
De Pal.ma, G. et al.	2012	E (abstract)	A	Y – Abstract – Not relevant
De Pal.ma, G. et al.	2014	E (abstract)	A	Y – Abstract – Not relevant
De Pal.ma, G. et al.	2017	E	A&H	Y – Abstract – Not relevant
Desbonnet, L. et al.	2014	E	A	Y – Abstract – Not relevant
Dinan, T & Cryan, J	2017	R	A&H	Y – Abstract – no abstract
Dinan, T & Quigley, E.	2011	R	?	Y – Abstract – no abstract
Dinan, T et al.	2015	R	A&H	Y – Abstract – Not appropriate
Eiwegger, T et al.	2010	E	A	Y – Abstract – Not relevant
Elgazzar, O. et al.	2018	E	A	Y – Abstract – Not relevant
Evrensel & Ceylan	2015	R	?	Y – Abstract – Not relevant
Ezenwa, V et al.	2012	R	A	Y – Abstract – Not relevant
Farzaneh & Tafvizi	2018	R	A	Y – Abstract – not available
Feuz & Ait-Belgnaoui	2015	R	?	Y – Abstract – Not appropriate
Forsythe, P et al.	2016	R	A&H	Y – Abstract – Not appropriate
Foster, J	2015	R (abstract)	?	Y – Abstract – Not appropriate
Foster, J & Neufeld K-A	2013	R	?	Y – Abstract – Duplicate
Fröhlich, E et al.	2016	E	A	Y – Abstract – Duplicate
Ghosh, D	2018	R	?	Y – Abstract – Not appropriate
Goudarzvand, M et al.	2016	E	A	Y – Abstract – Not relevant
Gruber, K.	2012	R	?	Y – Abstract – not English
Gueveorkyan, A et al.	2018	E	A	Y – Abstract – Not relevant
Gullón, P. et al.	2014	E	n/a	Y – Abstract – Not appropriate
Hayley, S et al.	2016	R	A&H	Y – Abstract – Not relevant
Hemmings, S et al.	2016	E	H	Y – Abstract – Not relevant
Hsiao, E et al.	2013	E	A	Y – Abstract – Not relevant
Huynh, K. et al.	2016	R	?	Y – Abstract – Not relevant
Inserra, A et al.	2018	R	A&H	Y – Abstract – Not relevant

**Author**	**Year**	**Experiment / Review**	**Type** Human (H), animal. (A), n/a, or unknown (?)	**Excluded Y/N** (Level of exclusion and reason)
Jermy, A	2011	R (comment)	A	Y – Abstract – Not appropriate
John, N,	2014	R (comment)	H	Y – Abstract – Not appropriate
Kaelberer, M. & Bohorquez, D	2018	R (comment)	?	Y – Abstract – Not appropriate
Kamimura, Y & Lanier, L	2013	E	n/a	Y – Abstract – Not appropriate
Kang, Y & Cai, Y.	2017	R	?	Y – Abstract – Not appropriate
Kellman, R	2017	R (comment)	?	Y – Abstract – Not appropriate
Kelly, J	2015	E (abstract)	?	Y – Abstract – Not appropriate
Kelly, J. et al.	2016	E (abstract)	A	Y – Abstract – Not appropriate
Koopman, M & El Aidy, S.	2017	R	?	Y – Abstract – Not appropriate
Kramer, P. & Bressan, P.	2015	R	al.l	Y – Abstract – Not relevant
Kretzschmar, A	2017	R	?	Y – Abstract – not available
Kuti, D. et al.	2017	E (abstract)	A	Y – Abstract – not available
Lewis, S.	2011	R (comment)	A	Y – Abstract – Not appropriate
Liang, S. et al.	2018	R	A	Y – Abstract – Not appropriate
Lim, S-M et al.	2017	E	A	Y – Abstract – Not relevant
Lindner, C. et al.	2015	E	n/a	Y – Abstract – Not relevant
Luk, B. et al.	2018	E	A	Y – Abstract – Not relevant
Luo, J. et al.	2014	E	A	Y – Abstract – Not appropriate
Lyte, M.	2017	?	?	Y – Abstract – nil available
Lyte, M. et al.	2016	E	A	Y – Abstract – not relevant
Lyte, M. & Cryan, J	2014	?	?	Y – Abstract – nil available
Macedo, D. et al.	2017	R	?	Y – Abstract – not related
MacMillan, A	2017	R (comment)	A	Y – Abstract – grey lit
Magnano, M. et al.	2012	?	?	Y – Abstract – nil English
Mal.ick, M. et al.	2015	E	A	Y – Abstract – not related
Mason, B. et al.	2017	E (abstract)?	?	Y – Abstract – nil available
McLean, P. et al.	2012a	E (abstract)?	?	Y – Abstract – nil available
McLean, P. et al.	2012b	E (abstract)?	?	Y – Abstract – nil available
Mediavilla, C. et al.	2015	E (abstract)	A	Y – Abstract – nil available
Meyer, C. & Vassar, M.	2018	R	H	Y – Abstract – nil available
Mika, A. et al.	2018	E	A	Y – Abstract – juvenile
Misheneva, V. et al.	2018	E (abstract)	A	Y – Abstract – nil available
Misra, S & Medhi, B	2013	?	?	Y – Abstract – letter/comment
Morris, A.	2018	R (comment)	A	Y – Abstract – comment
Murray, E.	2016	E (abstract)?	?	Y – Abstract – nil available
O'Mahony, S. et al.	2016	E	A	Y – Abstract – nil available
Pace, R. et al.	2018	E (abstract)	A	Y – Abstract – nil available
Panduro, A. et al.	2017	R	?	Y – Abstract – gut disorders
Parikh, I. et al.	2017	E (abstract)	A	Y – Abstract – nil available
Park, A. et al.	2013	E	A	Y – Abstract – not related
Park, C. et al.	2018	R	?	Y – Abstract – immune focus
Pinto-Sanchez, M. et al.	2017	E	H	Y – Abstract – IBS
Pop, O.L. et al.	2016	E	n/a	Y – Abstract – food
Proctor, C. et al.	2017	R	H&A	Y – Abstract – diet
Pyndt Jørgensen, B, et al.	2014	E	A	Y – Abstract – food
Rackers, H.S. et al.	2018	R	?	Y – Abstract – perinatal.
Rios. A. et al.	2017	R	A&H	Y – Abstract – not related

**Author**	**Year**	**Experiment / Review**	**Type** Human (H), animal. (A), n/a, or unknown (?)	**Excluded Y/N** (Level of exclusion and reason)
Robertson. R.C. et al.	2017	E	A	Y – Abstract – ω-3 interaction
Said, A.	2013	?	?	Y – Abstract – nil Eng
Sampson, R & Mazmanian, K	2015	R	A	Y – Abstract – perinatal.
Sanchez, M et al.	2017	E	H	Y – Abstract – obesity
Savingnac, H. et al.	2016	E	A	Y – Abstract – Not appropriate
Savingnac, H. et al.	2016	E	A	Y – Abstract – duplicate
Schachterm J. et al.	2018	R	?	Y – Abstract – obesity+psych
Schmidt, C.	2015	?	?	Y – Abstract – nil available
Seo, Y.C. et al.	2012	E	A	Y – Abstract – Not appropriate
Sharon, G. et al.	2016	R	Al.l	Y – Abstract – broad review
Shropshire, J. et al.	2016	R	?	Y – Abstract – Not appropriate
Slykerman, R. et al.	2018	E	H	Y – Abstract – childhood
Smith, A.P. et al.	2015	E	H	Y – Abstract – duplicate
Smythies, L. et al.	2014	R	?	Y – Abstract – no abstract
Spanier, R.	2018	R (abstract)	?	Y – Abstract – short comm.
Spasova, D. & Surh, C.	2013	R (abstract)	?	Y – Abstract – short comm.
Springen, K.	2017	?	?	Y – Abstract – grey lit
Stefaniak, A. et al.	2018	?	?	Y – Abstract – nil Eng
Stilling, R. et al.	2015	E	A	Y – Abstract – child developmt
Takajo, T. et al.	2017	E	A	Y – Abstract – abstract only
Thursby, E. & Juge, N.	2017	R	?	Y – Abstract – not relevant
Tillman, S. et al.	2017	E (abstract)	A	Y – Abstract – not relevant
Timmis, K. et al.	2017	?	?	Y – Abstract – editorial.
Tochitani, S. et al.	2016	E	A	Y – Abstract – paeds
Torres, E.R.S. et al.	2018	E	A	Y – Abstract – Parkinson's
Tottenham, N, et al.	2017	?	?	Y – Abstract – no abstract
Toyoda, A. et al.	2017	E	A	Y – Abstract – no abstract
van Baarlen, P et al.	2011	E	n/a	Y – Abstract – cellular
Van Hemert, S. et al.	2015	R (abstract)	A&H	Y – Abstract – psych focus
Van Hemert, S. et al.	2016	R (abstract)	?	Y – Abstract – psych focus
Vitetta, L. et al.	2014	R	?	Y – Abstract – commentary
Vlainić, J. et al.	2016	R	?	Y – Abstract – not relevant
Vuong, H.E. et al.	2017	R	?	Y – Abstract – not relevant
Wal.lace, C. et al.	2018	E (abstract)		Y – Abstract – not relevant
Wal.lace, C & Milev, R.	2017	R	H	Y – Abstract – Incorrect excl
Wal.lace, C. & Milev, R.	2017	R	H	Y – Abstract – erratum
Waworuntu, R. et al.	2014a	E (abstract)	A	Y – Abstract – development
Waworuntu, R. et al.	2014b	E (abstract)	A	Y – Abstract – development
Waworuntu, R. et al.	2014c	E (abstract)	A	Y – Abstract – development
Winter, G. et al.	2018	R	A&H	Y – Abstract – Not appropriate
Wong, M. et al.	2016	E	n/a	Y – Abstract – cellular
Xie, P.	2017	E (abstract)	A	Y – Abstract – mol. Pathway
Xinyan, P. et al.	2014	E	A	Y – Abstract – aging
Xu, N. et al.	2018	E	A	Y – Abstract – gut disease
**Relevent but excluded:**				
Animal. Studies				
Abdrabou, A. M. et al.	2018	E	A	Y – Abstract – animal.

**Author**	**Year**	**Experiment / Review**	**Type** Human (H), animal. (A), n/a, or unknown (?)	**Excluded Y/N** (Level of exclusion and reason)
Abildgaard, A. et al.	2017a	E	A	Y – Abstract – animal.
Abildgaard, A. et al.	2017b	E	A	Y – Abstract – animal.
Ait-Belgnaoui, A. et al.	2012	E	A	Y – Abstract – animal.
Avolio, E. et al.	2019	E	A	Y – Abstract – animal.
Bangsgaard Bendtsen et al.	2012	E	A	Y – Abstract – Animal.
Beilharz, J. E. et al.	2018	E	A	Y – Abstract – animal.
Bercik, P. et al.	2011	E	A	Y – Abstract – animal.
Bharwani, A. et al.	2016	E	A	Y – Abstract – animal.
Bharwani, A. et al.	2017	E	A	Y – Abstract – animal.
Bravo, J. A. et al.	2011	E	A	Y – Abstract – animal.
Burokas, A. et al.	2017	E	A	Y – Abstract – animal.
Campos, A. C. et al.	2016	E	A	Y – Abstract – animal.
Ceylani, T. et al.	2018	E	A	Y – Abstract – animal.
Crumeyrolle-Arias, M. et al.	2014	E	A	Y – Abstract – animal.
D'Mello, C. et al.	2015	E	A	Y – Abstract – animal.
Davari, S. et al.	2013	E	A	Y – Abstract – animal.
Davis, D. J. et al.	2017	E	A	Y – Abstract – animal.
De Pal.ma, G. et al.	2014	E	A	Y – Abstract – animal.
Denou, E. et al.	2011	E (abstract)	A	Y – Abstract – animal.
Desbonnet, L. et al.	2015a	E	A	Y – Abstract – animal.
Desbonnet, L. et al.	2015b	E	A	Y – Abstract – animal.
Dhal.iwal., J. et al.	2018	E	A	Y – Abstract – animal.
Distrutti, E. et al.	2014	E	A	Y – Abstract – animal.
Ermolenko, E. I. et al.	2018	E	A	Y – Abstract – animal.
Erny, D. et al.	2015	E	A	Y – Abstract – animal.
Fox, J. H. et al.	2017	E	A	Y – Abstract – animal.
Frank, M. G. et al.	2018	E	A	Y – Abstract – animal.
Fröhlich, E. E. et al.	2016	E	A	Y – Abstract – animal.
Gacias, M. et al.	2016	E	A	Y – Abstract – animal.
Gronier, B. et al.	2018	E	A	Y – Abstract – animal.
Guida, F. et al.	2018	E	A	Y – Abstract – animal.
Hoban, A. E. et al.	2016	E	A	Y – Abstract – animal.
Hoban, A. E. et al.	2017a	E	A	Y – Abstract – animal.
Hoban, A. E. et al.	2017b	E	A	Y – Abstract – animal.
Hoban, A. E. et al.	2018	E	A	Y – Abstract – animal.
Huo, R. et al.	2017	E	A	Y – Abstract – animal.
Jaglin, M. et al.	2018	E	A	Y – Abstract – animal.
Jang, H.-M, Lim, S. et al.	2018a	E	A	Y – Abstract – animal.
Jang, H.-M., Lee, K.-E. et al.	2008b	E	A	Y – Abstract – animal.
Janik, R. et al.	2016	E	A	Y – Abstract – animal.
Jung, I. H. et al.	2012	E	A	Y – Abstract – animal.
Kantak, P. A. et al.	2014	E	A	Y – Abstract – animal.
Kelly, J. R. et al.	2016	E	A	Y – Abstract – animal.
Kish, L. et al.	2011	E (abstract)	A	Y – Abstract – animal.
Lee, H. J., Jeong, J. J. et al.	2018a	E	A	Y – Abstract – animal.
Lee, H. J., Kim, D. H. & Hwang, Y. H.	2018b	E	A	Y – Abstract – animal.

**Author**	**Year**	**Experiment / Review**	**Type** Human (H), animal. (A), n/a, or unknown (?)	**Excluded Y/N** (Level of exclusion and reason)
Lee, H. J., Kim, D. H. and Lim, S. M.	2018c	E	A	Y – Abstract – animal.
Liang, S. et al.	2015	E	A	Y – Abstract – animal.
Liu, W.-H. et al.	2016	E	A	Y – Abstract – animal.
Loupy, K. M. et al.	2018	E	A	Y – Abstract – animal.
Luo, Y. et al.	2018	E	A	Y – Abstract – animal.
Lyte, M. et al.	2016	E	A	Y – Abstract – animal.
Magnusson, K. R. et al.	2015	E	A	Y – Abstract – animal.
Marin, I. A. et al.	2017	E	A	Y – Abstract – animal.
Matthews, D. M. & Jenks, S. M.	2013	E	A	Y – Abstract – animal.
McVey Neufeld, K. A.	2015	E	A	Y – Abstract – animal.
McVey Neufeld, K. A.	2017	E	A	Y – Abstract – animal.
McVey Neufeld, K. A.	2018	E	A	Y – Abstract – animal.
Neufeld, K. A. et al.	2011a	E	A	Y – Abstract – animal.
Neufeld, K. A. et al.	2011b	E	A	Y – Abstract – animal.
Nishino, R. et al.	2013	E	A	Y – Abstract – animal.
Ogbonnaya, E. S. et al.	2015	E	A	Y – Abstract – animal.
Ohland, C. L. et al.	2013	E	A	Y – Abstract – animal.
Ohsawa, K. et al.	2015	E	A	Y – Abstract – animal.
Payet, J. M. et al.	2018	E	A	Y – Abstract – animal.
Perez-Burgos, A. et al.	2013	E	A	Y – Abstract – animal.
Perez-Burgos, A. et al.	2014	E	A	Y – Abstract – animal.
Savignac, H. M. et al.	2013	E	A	Y – Abstract – animal.
Savignac, H. M. et al.	2015	E	A	Y – Abstract – animal.
Siebler, P. H. et al.	2018	E	A	Y – Abstract – animal.
Tarr, A. J. et al.	2015	E	A	Y – Abstract – animal.
Tillmann, S. et al.	2018	E	A	Y – Abstract – animal.
Tillman, S and Wegener, G.	2018	E	A	Y – Abstract – animal.
Vicentini, F. A. et al.	2018	E	A	Y – Abstract – animal.
Wang, T. et al.	2015	E	A	Y – Abstract – animal.
Winther, G. et al.	2015	E	A	Y – Abstract – animal.
Zeng, L., Zeng, B. et al.	2016	E	A	Y – Abstract – animal.
Narrative review or grey lit.				
Al.len. A. P. et al.	2017	R	H	Y – Abstract – narrative
Al.per, E. & Mehmet Emin, C.	2017	R	H	Y – Abstract – narrative
Anderson, S. C. et al.	2017	R	H	Y – Abstract – narrative
Archie, E. A. & Tung, J.	2015	R	H	Y – Abstract – narrative
Arcidiacono, S. et al.	2018	R	Al.l	Y – Abstract – narrative
Baily, M. T. & Cryan, J. F.	2017	R	A&H	Y – Abstract – narrative
Barrett, E. et al.	2013	R	?	Y – Abstract – narrative
Bienenstock, J. et al.	2013	R		Y – Abstract – narrative
Borre, Y. E. et al.	2014	R		Y – Abstract – narrative
Can probiotics al.leviate stress and mood disorders?	2013	R	grey	Y – Abstract – narrative

**Author**	**Year**	**Experiment / Review**	**Type** Human (H), animal. (A), n/a, or unknown (?)	**Excluded Y/N** (Level of exclusion and reason)
Cawthon, C. R. & de La Serre, C. B.	2018	R		Y – Abstract – narrative
Collins, Kassam, Bercik	2013	R		Y – Abstract – narrative
Cryan, J. F. & Clarke, G.	2016	R		Y – Abstract – narrative
Cryan, J. F. & Dinan, T. G.	2012	R		Y – Abstract – narrative
Cryan, J. F. & Dinan, T. G.	2015	R		Y – Abstract – narrative
Cryan, J. F. & Dinan, T. G.	2019	R		Y – Abstract – narrative
Cryan, J. F. & O'Mahony, S. M.	2011	R		Y – Abstract – narrative
Dash, S. et al.	2015	R		Y – Abstract – narrative
Davidson, G. L.	2018	R		Y – Abstract – narrative
Dinan, T. G. & Cryan, J. F.	2013	R		Y – Abstract – narrative
Dinan, T. G. & Cryan, J. F.	2015	R		Y – Abstract – narrative
Dinan, T. G. & Cryan, J. F.	2017a	R		Y – Abstract – narrative
Dinan, T. G. & Cryan, J. F.	2017b	R		Y – Abstract – narrative
Dinan, T. G., et al.	2013	R		Y – Abstract – narrative
Donovan, S. M.	2017	R		Y – Abstract – narrative
Forsythe, P. et al.	2016	R		Y – Abstract – narrative
Foster, J. A.	2016	R		Y – Abstract – narrative
Frank, M. G. et al.	2018	R		Y – Abstract – narrative
Gareau, M. G.	2014	R		Y – Abstract – narrative
Gareau, M. G.	2016	R		Y – Abstract – narrative
Gayathri, D. & Rashmi, B. S.	2017	R		Y – Abstract – narrative
Gilbert, J. A. et al.	2018	R		Y – Abstract – narrative
Glaven, S. et al.	2018	R		Y – Abstract – narrative
Goldman, E.	2018	R		Y – Abstract – narrative
Greener, M.	2018	R		Y – Abstract – narrative
Gulati, G. & Mulryan, D.	2018	R		Y – Abstract – narrative
Heijtz, R. D. et al.	2011	R		Y – Abstract – narrative
Johnson, K & Foster, K.	2018	R		Y – Abstract – narrative
Kane, L & Kinzel, J.	2018	R		Y – Abstract – narrative
Karakula-Juchnowicz, H. et al.	2016	R		Y – Abstract – narrative
Kelly, J. R. et al.	2015	R		Y – Abstract – narrative
Kuo, P. H & Chung, Y. C. E.	2018	R		Y – Abstract – narrative
Langgartner, D. et al.	2019	R		Y – Abstract – narrative
Lewis, A.	2017	R		Y – Abstract – narrative
Licinio, J. et al.	2017	R		Y – Abstract – narrative
Lima-Ojeda, J. M. et al.	2017	R		Y – Abstract – narrative
Liu, L. & Zhu, G.	2018	R		Y – Abstract – narrative
Luczynski, P. et al.	2016a	R		Y – Abstract – narrative
Luczynski, P. et al.	2016b	R		Y – Abstract – narrative
Luna, R. A. & Foster, J. A.	2015	R		Y – Abstract – narrative
Lyte, M.	2013	R		Y – Abstract – narrative
Lyte, M.	2014	R		Y – Abstract – narrative
Mal.an-Muller, S. et al.	2018	R		Y – Abstract – narrative
Mohajeri, M. H. et al.	2018	R		Y – Abstract – narrative
Montiel-Castro, A. J. et al.	2013	R		Y – Abstract – narrative

Author	Year	Experiment / Review	Type Human (H), animal. (A), n/a, or unknown (?)	Excluded Y/N (Level of exclusion and reason)
Münger, E. et al.	2018	R		Y – Abstract – narrative
Noble, E. et al.	2017	R		Y – Abstract – narrative
Ntranos, A & Casaccia, P.	2018	R		Y – Abstract – narrative
O'Mahony, S. M. et al.	2015	R		Y – Abstract – narrative
Oaklander, M.	2015	R	Grey	Y – Abstract – narrative
Osadchiy, V. et al.	2018	R		Y – Abstract – narrative
Parashar, A. & Udayananu, M.	2016	R		Y – Abstract – narrative
Prebiotics may help ease (grey)	2015	R		Y – Abstract – narrative
Probiotics may help address (grey)	2015	R		Y – Abstract – narrative
Pusceddu, M, M.	2018	R		Y – Abstract – narrative
Rogers, G. B.	2016	R		Y – Abstract – narrative
Sarkar, A.	2016	R		Y – Abstract – narrative
Sarkar, A,	2018	R		Y – Abstract – narrative
Selhub, E. M.	2014	R		Y – Abstract – narrative
Stamper, C. E. et al.	2016	R		Y – Abstract – narrative
Stilling, R. M. et al.	2014a	R		Y – Abstract – narrative
Stilling, R. M. et al.	2014b	R		Y – Abstract – narrative
Sylvia, K. E. & Demas, G. E.	2018	R		Y – Abstract – narrative
Taverniti, V. & Guglielmetti, S.	2011	R		Y – Abstract – narrative
Waclawiková, B. & El Aidy, S.	2018	R		Y – Abstract – narrative
Systematic Literature Reviews				
Brenner, L. A. et al.	2017	R	H	Y – Abstract – SLR
Clark, A. & Mach, N.	2016	R	H	Y – Abstract – SLR
Huang, R. et al.	2016	R	H	Y – Abstract – SLR
Liu, B, et al.	2018	R	H	Y – Abstract – SLR – probiotics
Ng, Q. X. et al.	2018	R	H	Y – Abstract – SLR – probiotics
Pirbaglou, M, et al.	2016	R	H	Y – Abstract – SLR – probiotics
Romijn, A. R. & Rucklidge, J. J.	2015	R	H	Y – Abstract – SLR
Slyepchenko, A. et al.	2014	R	H	Y – Abstract – SLR – probiotics
Slyepchenko, A. et al.	2017	R	H	Y – Abstract – SLR
Wang , H. et al.	2016	R	H&A	Y – Abstract – SLR
Excluded Full Review				
Akkasheh, G. et al.	2016	E	H	Y – Full – depression
Cepeda, M. S. et al.	2017	E	H	Y – Full – depression
Colica, C. et al.	2017	E	H	Y – Full – stress & anxiety
Culpepper, T. et al.	2016	E	H	Y – Full – stress & anxiety
Kazemi, A. et al.	2018	E	H	Y – Full – depression
Messaoudi, M. et al.	2011a	E	H	Y – Full – stress & anxiety
Messaoudi, M. et al.	2011b	E	H	Y – Full – stress & anxiety

**Author**	**Year**	**Experiment / Review**	**Type** Human (H), animal. (A), n/a, or unknown (?)	**Excluded Y/N** (Level of exclusion and reason)
Mohammadi, A. A. et al.	2016	E	H	Y – Full – stress & anxiety
Murata, M. et al.	2018	E	H	Y – Full – stress & anxiety
Nishida, K. et al.	2017	E	H	Y – Full – stress & anxiety
Nishihira, J. et al.	2014	E	H	Y – Full – stress & anxiety
Noorwal.i, E. A. et al.	2017	E	H	Y – Full – stress & anxiety
Owen, L. et al.	2014	E (abstract)	H	Y – Full – < 2017
Perez-Cornago, A. et al.	2016	E	H	Y – Full – depression
Romijn, A. R. et al.	2017	E	H	Y – Full – depression & anxiety
Sawada, D. et al.	2017	E	H	Y – Full – stress & anxiety
Shinkai, S. et al.	2013	E	H	Y – Full – immune only
Slykerman, R. F. et al.	2017	E	H	Y – Full – depression & anxiety
Steenbergen, L. et al.	2015	E	H	Y – Full – mood
Takada, M. et al.	2016	E	H&A	Y – Full – stress
Takada, M. et al.	2017	E	H	Y – Full – environmental. stress
Tal.lbot, S. et al.	2018	E (abstract)	H	Y – Full – multi-psych

List of abbreviations: Experiement (E); Review (R); Human (H), Animal. (A), not applicable (n/a); unknown (?); yes (Y); systematic literature review (SLR)
